# Larynx cancer mortality in the State of Pernambuco-Brazil-2000-2004

**DOI:** 10.1016/S1808-8694(15)30781-3

**Published:** 2015-10-19

**Authors:** Leandro de Araújo Pernambuco, Mirella Bezerra Rodrigues Vilela

**Affiliations:** 1 Graduate Student of Dysphagia in the Faculdade Integrada do Recife - FIR. Speech and Hearing Therapist - Hospital de Câncer de Pernambuco and Centro de Reabilitação e Fisioterapia Distrito I of Jaboatão dos Guararapes city- PE; 2 MSc in Public Health - Centro de Pesquisas Aggeu Magalhães - Fundação Oswaldo Cruz. Substitute Professor - Speech and Hearing Therapy - Undergraduate Program - Universidade Federal de Pernambuco and Head of the Health Care Information - Prefeitura Municipal do Jaboatão dos Guararapes - PE. Universidade Federal de Pernambuco

**Keywords:** epidemiology, mortality, laryngeal cancer

## Abstract

The larynx is considered a site of the greatest occurrence of head and neck neoplasias, and for cancer studies, mortality is one of the most reliable health indicators.

**Aim:**

to study the mortality by laryngeal cancer in Pernambuco during 2000–2004.

**Study format:**

contemporary cross-sectional cohort.

**Materials and Methods:**

we considered all deaths by laryngeal cancer in residents of Pernambuco State between 2000 and 2004, taken from the State's Mortality Information System (SIM/SUS). The data was analyzed through descriptive statistics, with the results expressed in tables, graphs and maps, using Excel version 2000 and the EpiInfo version 6.04b software.

**Results:**

There was little variation in the mortality coefficient in the years considered for study. The Sertão Mesoregion had the highest number of deaths and Fernando de Noronha island had the highest mortality rate. The patient profile found was: men, between 60–69 years, brown color, married, with low literacy, who died in a hospital setting.

**Conclusion:**

we found mortality stability and heterogeneity among the cities. The mortality profile according to social variables corroborates data found in other Brazilian States, except for race/color.

## INTRODUCTION

Following the period of global reorganization that happened in the past century, public health entered a process called epidemiological transition^1^. Such phenomenon is basically characterized by three events: a drop in mortality rates by infectious and parasitic diseases (IPD) together with an increase in the number of non-transmittable diseases and disorders (NTDD), as well as cardiovascular diseases and cancer; transmission of the morbi-mortality load in the younger population to the elderly; change in the mortality dominance profile towards a profile where morbidity prevails^2^.

In developed countries, this epidemiologic transition has been completed, however it is still ongoing in developing countries. In Brazil, as well as in other Latin American countries, se see that there is an increase in the number of deaths by NTDD; however, mortality rates by IPDs still remain a public health problem^1^.

Today, cancer represents the third largest cause of male deaths in Brazil, right after cardiovascular diseases and external causes. As we consider both genders and individuals above forty years of age, cancer takes the second place as cause of death, being second only to cardiovascular disorders^3^, ^4^, ^5^. Laryngeal cancer is the number one head and neck cancer and represents the second most common type of respiratory cancer in the world, second only to lung cancer^6^, ^7^, ^8^, ^9^. This type of neoplasia represents 2.8% of the new cases of cancer in men in the world, corresponding to the tenth most frequent malignant neoplasia in men^7^.

Despite risk factor diversity, smoking and drinking are considered the main etiologies^10^.

In Pernambuco, the mortality coefficient for laryngeal cancer in men during 1979/1981 was of 0.8/100,000, while during 1999/2001 this coefficient was raised to 1.9/100,000^11^.

Mortality rates contribute substantially for most of the other health care indicators, representing the main index used to characterize the importance of cardiovascular diseases and malignant neoplasia. Even in more developed countries, where it is relatively easy to obtain morbidity data, mortality rates remain one of them most important, if not the most important, health care indicator^12^.

The Brazilian Department of Health posts in the Internet, by means of DATASUS, information that can serve as a tool for making an objective analysis of the health situation, evidence-based decision making and to program health care actions in Brazil. From DATASUS one can obtain a vast array of information regarding systematic data records on mortality; survival; infectious, parasitic and chronic diseases; data on morbidity, disability, access to services; health care quality, life conditions and environmental factors; information on the health care provided to the population, records from hospitals' networks, outpatient wards and health care facilities, and also information about financial resources systems and demographic and socio-economic information^13^.

Thus, we can notice that this tool allows us to study mortality, which can guide approaches and serve as a basis to plan strategic actions. This way, the goal of the present paper was to study the development of laryngeal cancer-related deaths in the State of Pernambuco during the 2000–2004 years, and the comparative prevalence among its cities and regions, as well as to trace the predominant profile of dying patients.

## METHODS

The study area was made up of the state of Pernambuco, divided in its 185 towns. The State's towns are politico-administratively gathered into five meso-regions, namely: São Francisco, Sertão, Agreste, Zona da Mata and Metropolitana do Recife, and also the Distrito Estadual de Fernando de Noronha. The study population was made up of all the deceased people who resided in the state of Pernambuco, who died during 2000 to 2004, because of laryngeal cancer, classified by means of the 10th Edition of the International Classification of Diseases (ICD) - code C32.

The data was collected through information made available by the Department of Health in its Web Site: DATASUS^14^, by means of the Information System on Mortality (SIM/SUS). Population data was obtained through the IBGE, using the National Census of 2000, and the population estimates for the intermediate years by means of National Surveys of Population Samples (NSPS), led annually by the IBGE. The variables considered in this study were: year, gender, age, marital status, literacy, race/color and place of occurrence.

This is a contemporary cross-sectional cohort study. We did not included assisted patients; therefore, it was not necessary to submit this paper to the ethics committee. We have preserved the right of source disclosure (SIM/SUS and IBGE), there was no damage to the individuals' health, considering that all data collection was carried out in a secondary basis.

Data analysis was carried out through a descriptive analysis with its results expressed in tables, graphs and a map. For that, we used Microsoft Excel version 2000 software and the EpiInfo, version 6.04b, produced by the Center for Disease Control and Prevention (CDC). As an association measure, we used the relative risk (RR), confirmed through the Yates-corrected chi-squared statistical methodology, with a statistical significance of 5% (p< 0.05).

For data mapping, we used the mortality coefficient standardized formula^15^, which is based on dividing the number of deaths by laryngeal cancer in a specific area, during the analysis period, by the resident population in that area and in the same period, multiplied by 100,000. The theme map was created on the Terraview, version 3.1.4 software, produced by the INPE (Instituto Nacional de Pesquisas Espaciais - The National Institute of Space Research).

## RESULTS

Between 2000 and 2004, in the State of Pernambuco, 452 deaths by laryngeal cancer were recorded, representing 2.61% of the total deaths caused by a malignant neoplasia.

**Graph 1:**
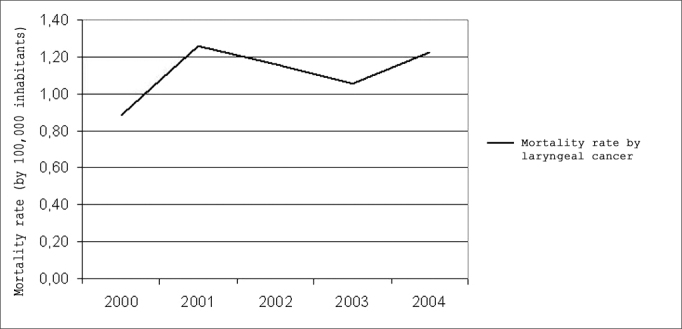
Mortality rate by laryngeal malignant neoplasia in Pernambuco, 2000 – 2004.

Source: Mortality Information System /Health Department.

In Graph 1, it is possible to analyze the development of laryngeal cancer in Pernambuco. The mortality coefficients show that there was no significant variation in the years studied.

**Figure 1:**
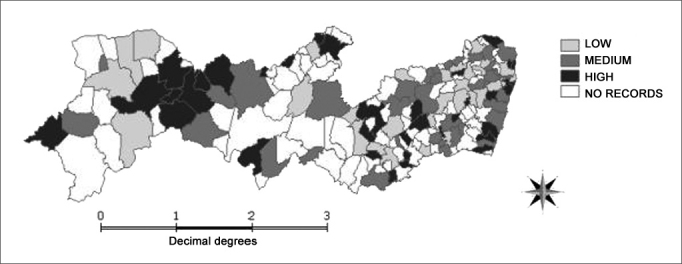
Distribution of deaths by laryngeal malignant neoplasia in Pernambuco, according to city of residence, 2000–2004.

Source: Mortality Information System /Health Department; Terraview/National Institute of Space Research.

Analyzing mortality by laryngeal cancer in the 185 towns of the state of Pernambuco in the period between 2000 and 2004, we noticed that 103 cities, that is 55.7%, had death records associated with this cause (Figure 1).

As these cities were grouped by mesoregions, it was observed that the Sertão region was the one that had the clusters with the highest mortality coefficients when compared to the others, made up by the towns of São José do Belmonte, Verdejante, Salgueiro, Cabrobó, Terra Nova, Serrita and Parnamirim.

Besides these five towns, fifteen others presented high mortality coefficients. They were: Ilha de Itamaracá, Itapissuma, Recife, Olinda and Moreno (Metropolitan mesoregion); Rio Formoso, Barreiros, Chã Grande and Itambé (Zona da Mata mesoregion); São Caetano, Altinho, Lajedo, Angelim and Palmeirinha (Agreste mesoregion); Afrânio (Vale do São Francisco mesoregion). The island of Fernando de Noronha was the place that had the highest mortality coefficient in the state (46.9/100,000) (Figure 1).

**Graph 2:**
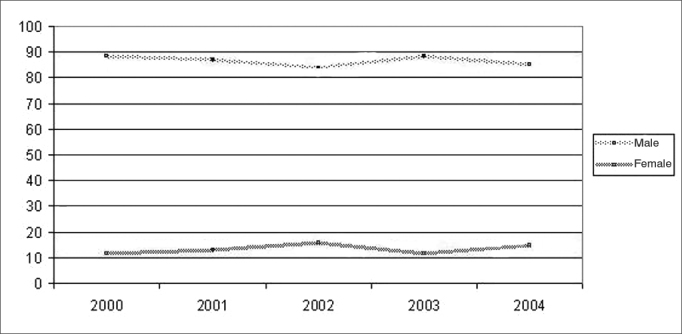
Mortality by laryngeal cancer in Pernambuco, according to gender, 2000–2004.

Source: Mortality Information System /Health Department.

When we studied gender, we noticed that during the period studied, male rates were higher than the female ones (Graph 2). The relative risk of death by laryngeal cancer was 6.85 fold higher for men when compared to women (5.23 < RR < 8.98), and this data was statistically significant (X 2=262; p< 0.001).

Analyzing data regarding mortality rates according to age range (Table 1), we observed that the individuals whose age varied between 60 and 69 years of age made up the group with the highest death rates (28.10%).

**Table 1:** Death rates by laryngeal cancer in Pernambuco, broken down by age during 2000–2004.

	2000		2001		2002		2003		2004		TOTAL	
Age	N	%	N	%	N	%	N	%	N	%	N	%
15 to 19 years	1	1.43	0	0.00	0	0.00	0	0.00	0	0.00	1	0.22
30 to 39 years	1	1.43	2	1.98	0	0.00	1	1.16	1	0.99	5	1.11
40 to 49 years	6	8.57	16	15.84	10	10.64	6	6.98	9	8.91	47	10.40
50 to 59 years	11	15.71	22	21.78	21	22.34	20	23.26	19	18.81	93	20.58
60 to 69 years	20	28.57	27	26.73	30	31.91	23	26.74	27	26.73	127	28.10
70 to 79 years	22	31.43	19	18.81	20	21.28	25	29.07	30	29.70	116	25.66
80 years and older	9	12.86	15	14.85	13	13.83	11	12.79	15	14.85	63	13.94
Total	70	100.00	101	100.00	94	100.00	86	100.00	101	100.00	452	100.00

As they are compared to the group of patients between 15 and 19 years, with the lowest rates among all the groups (0.22%), those who were in the age range between 60 and 69 years had a relative risk 293 fold higher of dying of laryngeal cancer (41.01 < RR < 299.05; X 2=285; p< 0.001). Now, when the relative risk was evaluated in relation to the age range between 50 and 59 years (20.58%), it dropped to 2.01 (1.54 < RR < 2.62; X 2=26.48; p< 0.001).

As for the race/skin color variable, we found among the age groups, a higher rate of deaths by laryngeal cancer among brown individuals (42.04%), followed by white individuals (37.39%) and blacks (9.29%). The rate of individuals classified as unknown was of 9.51%.

As to literacy rates (Table 2), we've noticed that the rate of records classified as unknown made up more than half of the sample in the group of years (52.21%) and in each year studied, alone. If we disregard the unknown ones, the group without any level of schooling (20.58%) and with 1 to 3 years of study (12.83%) represented the highest percentage of cases; however, this variable is not appropriate for analyses.

**Table 2:** Rate of deaths by laryngeal cancer in Pernambuco, according to education, 2000–2004.

	2000		2001		2002		2003		2004		TOTAL	
Schooling	N	%	N	%	N	%	N	%	N	%	N	%
None	17	24.29	17	16.83	16	17.02	16	18.60	27	26.73	93	20.58
1 to 3 years	11	15.71	17	16.83	10	10.64	9	10.47	11	10.89	58	12.83
4 to 7 years	2	2.86	10	9.90	13	13.83	7	8.14	7	6.93	39	8.63
8 to 11 years	1	1.43	6	5.94	3	3.19	2	2.33	3	2.97	15	3.32
12 years and more	2	2.86	1	0.99	3	3.19	3	3.49	2	1.98	11	2.43
Unknown	37	52.86	50	49.50	49	52.13	49	56.98	51	50.50	236	52.21
Total	70	100.00	101	100.00	94	100.00	86	100.00	101	100.00	452	100.00

Of the 452 deaths by laryngeal cancer which happened between 2000 and 2004 in Pernambuco, 51.11% happened to married individuals, followed by 24.34% of singles and 14.82% of widowers. In relation to the place of occurrence, we found a large predominance of deaths in the hospital (69.47%), followed by death at home (28.76%). The unknown information presented a very low rate (0.22%), corresponding to only one case.

## DISCUSSION

In Pernambuco, the mortality situation by laryngeal cancer seems to be identical to national figures, corresponding to 2.61% of all the deaths by cancer between 2000 and 2004^16^^,^^17^.

We did not have significant differences in the mortality rates by laryngeal cancer in the years studied, differently from a historic series carried out in Pernambuco, pointing towards an increase in this rate in all the mesoregions of the state between 1979 and 2001^11^. Despite the suggestive trend towards stability in the five years studied, this profile deserves attention, because it points towards a worsening in the situation that may lead to the development of higher rates in the future, especially when we consider the marked increase in the life expectancy of the population.

This same study carried out in Pernambuco between 1979 and 2001, also showed Sertão as the area of the largest concentration of deaths by malignant laryngeal neoplasia, corroborating data found in the present study. In 1979, the metropolitan mesoregion had the highest coefficient (1.8/100,000), while Sertão and Agreste areas shared the second place. As we reached 2001, this scenario reverted and the Sertão mesoregion became the place where the most deaths happened associated with the cause studied^11^.

One hypothesis that could justify the concentration of deaths in the Sertão mesoregion would be the difficulty of the population in having access to health care services, often times located far from their residences. Such situation causes a late diagnosis, with consequent disease progress and lower treatment efficacy. Thus, mortality rates tend to be higher.

This same hypothesis could also justify the fact that Fernando de Noronha Island has the highest mortality rate found in the study, because it is a place in which access to specialized health care is precarious, and where it is difficult to continue with the cancer treatment, the island becomes a place where the risk of death is higher. Moreover, the high local rate of alcohol consumption reflects the existence of an important risk factor for the development of malignant neoplasia.

We can also suppose that the heterogeneity among the records, as found in the state of Pará18, points towards possible differences in the quality of laryngeal neoplasia notification as basic cause of death. It is worth stressing that, despite the important improvements seen in health care information systems in Brazil, these still search for a broader data coverage and quality of the information generated^1^.

The data found calls for the need to decentralize oncologic services, allowing for the creation of diagnose and treatment centers in locations that need this type of service, aiming not only to provide access to the population to global treatment in their place of residence, but also cost reduction for the towns themselves, which end up spending an important amount of their government grant to invest in the transportation of patients to be treated outside their home areas.

As far as gender is concerned, males prevail in the literature. Western and Southern Europe are regions where mortality by laryngeal cancer among men have the highest rates of occurrence, followed by South American Countries such as Argentina, Uruguay and Southern Brazil^16^. In Porto Alegre, head and neck neoplasias (oral cavity, oropharynx, larynx and hypopharynx) they were the most frequent in men, considering the cases under treatment in the Centers of High Complexity in Oncology (CACON) of the town in 2000 and 2001^19^. Between 1979 and 1999, São Paulo was the state that had the highest mortality rates by laryngeal cancer in men (6.2/100,000)^20^. In Pará state, between 1980 and 1997 more men than women died because of this type of cancer^18^.

The world trend is for a reduction of deaths by laryngeal cancer in men and the stability in mortality rates among females, associating this fact to changes in the life style of women, such as an increase in the number of women smoking^17^. In the present investigation, we have seen a stability reaching both genders, in relation to the years studied. Such stability also happened in the first half of the 90's, in Brazil, for all types of tobacco-related cancers (mouth, pharynx, esophagus, pancreas, larynx, bladder and kidneys), except lung^4^ and in São Paulo, between 1969 and 1998^21^.

As far as age is concerned, we have seen that other studies also report a greater incidence of neoplasias on the fifth decade of life^5^^,^^10^^,^^16^^,^^17^^,^^22^. It is known that a raise in life expectancy increases the frequence of disorders and non-transmittable diseases, reflex of the epidemiological transition2. Although the mortality rate for laryngeal cancer has varied somewhat between 2000 and 2004, special attention must be given to the disease, considering its predominance in the elderly, and the accelerated pace of population aging. Because of this, we need to create policies to fight risk factors and bring about a healthy aging for the population.

Race/color is a variable that is not very often considered in studies carried out about laryngeal cancer. Very likely because in Northeastern Brazil we have more individuals classified as brown, we find in this race/skin color, a higher rate of deaths. In a town of the state of São Paulo, most of the patients with laryngeal cancer treated in a health care facility of the region were whites. In the United States, the greatest incidence among females is among African Americans16. The rate of individuals classified as unknown (race) was relatively high, considering that it is below that of browns and whites in the groupings of years. Nevertheless, we've seen a noticeable improvement in this variable, because in the year 2000 the rate of unknowns (17.14%) was much higher than the one found in 2004 (2.97%).

As far as schooling is concerned, we noticed that this variable is not reliable. Such statement is justified by the fact that the proportion of records classified as unknown make up more than half of the sample in the groups of years (52.21%) and, alone, in each year surveyed. Therefore, we see that the notification of this data seems to be a failure, and such fact could be generating biases among the results found. Disregarding the unknown ones, we noticed that the lower the schooling, the greater the risk of death by laryngeal cancer. Such data is in agreement with the literature^16^^,^^22^^,^^23^.

As far as marital status is concerned, the highest mortality rates found are among married individuals. The rates suffered mild alterations along the years surveyed; however, married individuals always kept the highest rates when compared to the others. Such result is in agreement with the records obtained through the analysis of death certificates in Rio de Janeiro in 1990 and 1999^25^^,^^26^. Just as schooling, marital status is considered an important factor impacting on the overall quality of life of individuals with head and neck cancer24. In the literature we did not find any reference making any sort of direct relation between death and the subject's marital status, and such data is more associated with quality of life.

The study stated the high percentage of hospital deaths, which can be justified because cancer belongs to a set of diseases that need specialized care. Considering the disease properties, the patient must be watched carefully in a hospital in order to monitor the neoplasia. Therefore, hospital deaths are more frequent than others, as stated in other studies^25^^,^^26^.

Reflecting on the results of the present investigation, we see that the information collected by this type of study is important in order to understand the epidemiological situation of a given region. The results found can serve as tools to seek improvements in health care, scope and quality of care for patients with malignant neoplasia in Pernambuco State. We suggest that studies like this one should also be carried out in other states and regions, in order to establish a more detailed and updated picture of cancer in Brazil.

## CONCLUSION

This study found a relatively stable profile for laryngeal cancer death in Pernambuco, in the five years between 2000 and 2004, suggesting that there is a trend for minimum annual variations. The differences among Pernambuco towns is in the distribution of deaths by laryngeal cancer, with a large concentration of deaths in the Sertão region and areas far from the state capital.

The social variables revealed a predominance of the following patient profile: males in their sixth decades of life, brown, married, low schooling, who died in a hospital setting.
